# Eliminating Aluminum Toxicity in an Acid Sulfate Soil for Rice Cultivation Using Plant Growth Promoting Bacteria

**DOI:** 10.3390/molecules20033628

**Published:** 2015-02-20

**Authors:** Qurban Ali Panhwar, Umme Aminun Naher, Othman Radziah, Jusop Shamshuddin, Ismail Mohd Razi

**Affiliations:** 1 Department of Land Management, Faculty of Agriculture, Universiti Putra Malaysia, UPM Serdang, Selangor 43400, Malaysia; 2 Soil Chemistry Section, Agricultural Research Institute, Tandojam 70060, Sindh, Pakistan; 3 Institute of Tropical Agriculture, Universiti Putra Malaysia, UPM Serdang, Selangor 43400, Malaysia; 4 Bangladesh Rice Research Institute, Gazipur 1701, Bangladesh

**Keywords:** Al speciation, chelation, indoleacetic acid, phytohormone, ameliorative effect

## Abstract

Aluminum toxicity is widely considered as the most important limiting factor for plants growing in acid sulfate soils. A study was conducted in laboratory and in field to ameliorate Al toxicity using plant growth promoting bacteria (PGPB), ground magnesium limestone (GML) and ground basalt. Five-day-old rice seedlings were inoculated by *Bacillus* sp., *Stenotrophomonas maltophila*, *Burkholderia thailandensis* and *Burkholderia seminalis* and grown for 21 days in Hoagland solution (pH 4.0) at various Al concentrations (0, 50 and 100 μM). Toxicity symptoms in root and leaf were studied using scanning electron microscope. In the field, biofertilizer (PGPB), GML and basalt were applied (4 t·ha^−1^ each). Results showed that Al severely affected the growth of rice. At high concentrations, the root surface was ruptured, leading to cell collapse; however, no damages were observed in the PGPB inoculated seedlings. After 21 days of inoculation, solution pH increased to >6.0, while the control treatment remained same. Field study showed that the highest rice growth and yield were obtained in the bio-fertilizer and GML treatments. This study showed that Al toxicity was reduced by PGPB via production of organic acids that were able to chelate the Al and the production of polysaccharides that increased solution pH. The release of phytohormones further enhanced rice growth that resulted in yield increase.

## 1. Introduction

Al toxicity is the main reason causing stunted root growth. In acidic soils, Al^3+^ limits the growth of roots either by inhibition of cell division, cell elongation or by both [1]. In rice and other cereals, this problem can cause about 30%–40% of crop yield reduction. Aluminum toxicity can be reduced by neutralizing the acidity using calcareous amendments [2]. Many plants have different mechanisms to tolerate the noxious effect of Al in response to this stress. These resistance mechanisms in plants have been categorized as: (a) external via the exudation of organic acids from the radical apexes and subsequent chelation of the Al in the rhizosphere; and (b) internal or Al-tolerant as Al chelation is produced inside the cell and then later stored and compartmentalized in cell organelles like the vacuole [3].

A small number of plant species have the ability to detoxify Al in the rhizosphere by exuding organic acids from their roots [4]. The exudation is situated in the radical apexes, as this is a place which is very susceptible to Al toxicity [5]. Organic acids play a vital role in external and internal neutralization of Al. Generally, organic acids secreted by plant roots are malate, citrate and oxalate. Malate and citrate are present in all cells which are required for the mitochondrial respiratory cycle [6]. The amount of organic acids released varies between plant species, and the detoxification mechanism is an internal tolerance [7,8,9]. Among the organic acids, malate is the one that reveals the least capacity to chelate Al ions [10].

It is proven that higher number of PGPR/PGPB is associated with rice rhizosphere [11] and they have the potential to produce a large amount of organic acids [12], which resulted in P binding by chelation, and may also be a possible mechanism for reducing Al toxicity of roots. The better performance of the PGPR/PGPB for the plant growth promotion occurs with the mixture of strains rather than individual strains [13]. In addition, the application of these beneficial microorganisms enhances the economic efficiency in terms of reduced production cost of phosphorus fertilizers [14]. Addition of these potential PGPR would enhance the growth of rice grown on soils with high Al content.

Low pH soils, especially acid sulfate soils, contain low total microorganisms, with their amount varying considerably according to vegetation type and soil management practices. Due to food security, attention is now focusing on rice production in less fertile acidic soils which are usually subjected to Al toxicity. Hence, the present study was undertaken: (1) to determine methods of increasing rice production in high Al containing soils using environmentally-friendly PGPB and/or soil amendments; and (2) to explain the possible mechanisms involved in this process.

## 2. Results and Discussion

### 2.1. Laboratory Study

**Figure 1:**
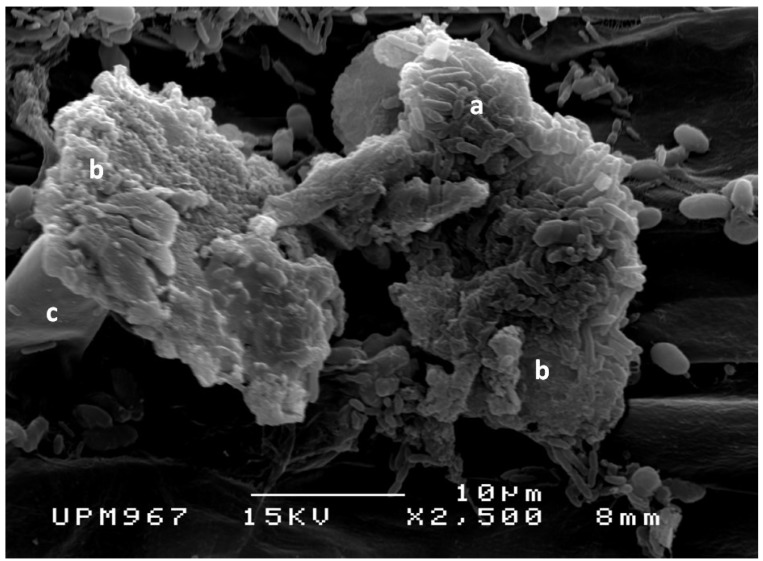
Exopolysaccharides production by the bacteria (**a**); showing gummy material (**b**) and covering root hair (**c**).

Various PGPB have been used in this study. These PGPB have the ability to fix nitrogen (*Stenotrophomonas maltophilla* Sb16), can solubilize phosphate and produce organic acids: (*Bacillus* sp. PSB16, *Burkholderia thailandensis*ASB7, and *Burkholderia seminalis* ASB21) (Table 1). All the bacteria were able to produce indoleacetic acid and exopolysaccharides in culture solution (Figure 1).

**Table 1:** Bio-chemical properties of the bacterial strains.

Strains	IAA (mg·L^−1^)	Production of Organic Acid (mg·L^−1^)				BNF	P solubilization from PR
		OA	MA	SA	PA		
*Bacillus* sp. (PSB16)	6.78	0.03	0.07	0.24	0.006	+ve	From soil * 86%
*Stenotrophomonasmaltophila* (Sb16)	55.00	0.06	0.04	0.39	0.008	** 62 kg·ha^−1^	-
*Burkholderiathailandensis* (ASB7)	13.16	0.02	0.05	0.24	0.012	+ve	From broth culture (72 h) 3.4%
*Burkholderiaseminalis* (ASB21)	12.16	0.09	0.08	0.42	0.018	+ve	From broth culture (72 h) 2.72%

Notes: IAA: Indoleacetic acid; OA: oxalic acid; MA: malic acid; SA: Salicylic acid; PA: propionic acid; BNF: biological nitrogen fixation; PR: phosphate rock. * determined using ^32^P isotope technique; ** determined using ^15^N isotope technique.

#### 2.1.1. Effect of Al on the Growth of Rice Seedlings Inoculated with PGPB

**Figure 2:**
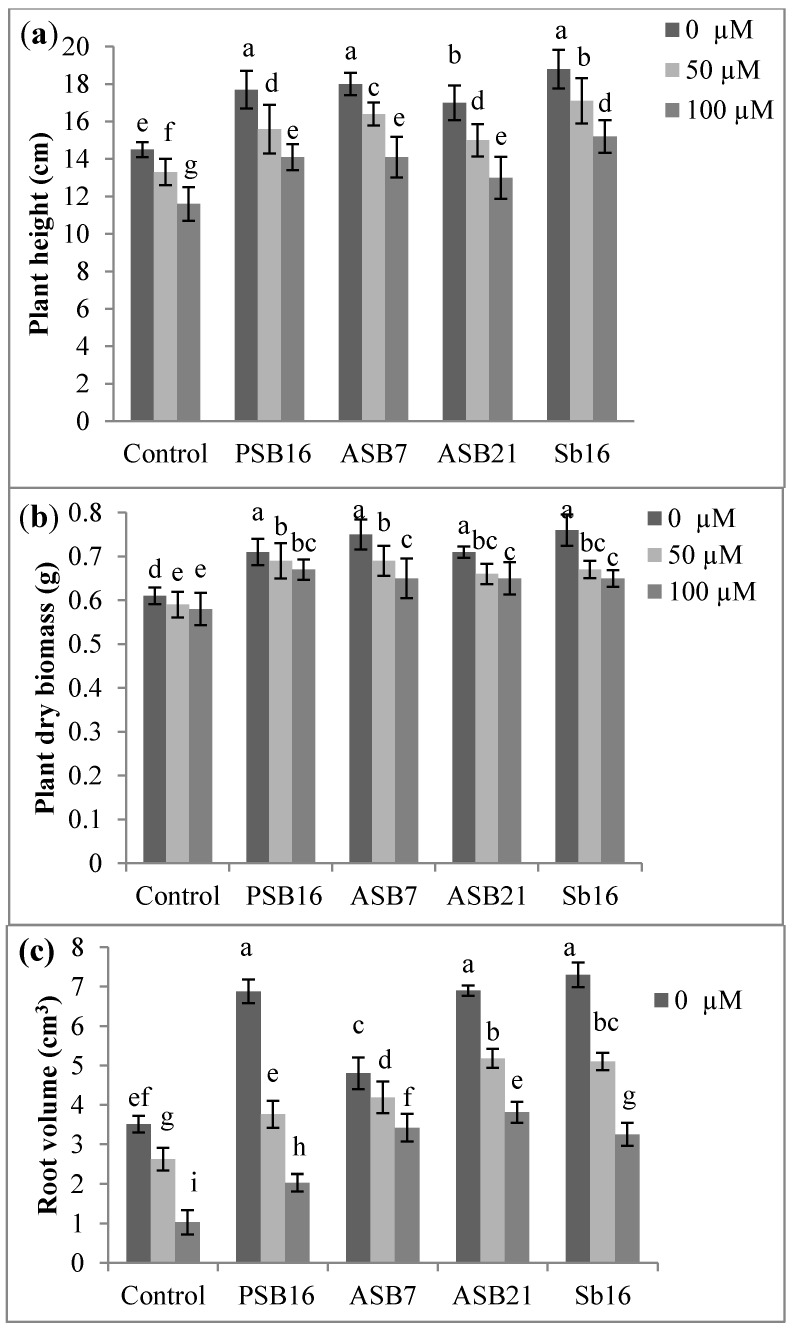
Effects of Al on the growth of rice seedlings inoculated with PGPB: (**a**) plant height; (**b**) plant dry biomass; and (**c**) root volume. Where, PSB 16 = *Bacillus* sp., Sb16 = *Stenotrophomonas maltophila*, ASB7 = *Burkholderia thailandensis*, ASB21 = *Burkholderia seminalis*. Means within the same column followed by the same letters are not significantly different at *p* < 0.05. Bars indicate standard error, *n* = 4.

It was observed that high Al concentration had severely affected the growth of the rice seedlings. Plant height, dry biomass and root volume were significantly decreased with the increased Al concentrations (Figure 2a–c). In contrast, the bacterial inoculated plants were less affected by the high Al concentration. The lowest plant height and dry biomass was found in the non-inoculated plants at 100 µM Al concentration. However, higher plant height (18.8 and 18 cm) and the highest plant dry biomass (0.76 and 0.75 g) were recorded due to Sb16 and ASB7 inoculation in the absence of Al. In this study, it was observed that root architecture and root volume varied with Al concentrations and bacterial inoculation. The presence of Al had affected roots and the detrimental effects were more profound at higher concentrations (Figure 2c). Generally, higher root volume was found in the inoculated compared to the non-inoculated rice plants. In the absence of Al, the highest root volume was obtained in the rice plant inoculated with Sb16 (7.3 cm^3^), followed by ASB21 (6.9 cm^3^) and PSB16 (6.88 cm^3^). The lowest root volume (1.03 cm^3^) was found in the control non-inoculated plants at 100 µM Al concentration. At the highest level of Al, higher root volume was observed in the inoculated rice plants compared to that of the control treatment.

#### 2.1.2. Effects of Al on the Population of PGPB

It was found that the population of PGPB was affected by the presence of Al. High Al concentration seemed to be slightly affected the PGPB population, shown by the higher population at 0 µM Al concentration compared to that of the others (Table 2). With the increase in Al concentration, the PGPB population decreased, and this trend was observed for all the PGPB strains. As the experiment was conducted in controlled conditions, there was no bacterial population recorded. However, there was a clear indication that bacterial strains still existed under the stress of low pH and high Al concentrations. Hence, these PGPB have the potential to be used in bio-fertilizer formulation for rice cultivation in acid sulfate soils.

**Table 2:** Effects of Al on the bacterial population and on pH of the growth medium.

Treatments	Bacterial Population (log_10_CFU·mL^−1^)			pH Values *		
	0	50	100	0	50	100
	Al (μM)					
Control	-	-	-	3.95c	3.25b	2.93c
*Bacillus* sp. (PSB16)	10.83b	9.23b	8.23b	7.12a	6.85a	6.50a
*Burkholderia thailandensis* (ASB7)	10.79b	9.68a	7.69c	6.85b	6.72a	6.64a
*Burkholderia seminalis* (ASB21)	10.87b	9.57a	8.11b	6.87b	6.65a	6.00b
*Stenotrophomonas maltophila* (Sb16)	11.07a	9.61a	8.54a	7.09a	6.71a	6.30a

Note: * The initial pH was fixed at 4.0. Means within the same column followed by the same letters are not significantly different at *p* < 0.05.

#### 2.1.3. Effects of PGPB on pH of the Growth Medium

It was observed that the pH of control treatment remained almost the same. On the other hand, in PGPB inoculated treatments, the pH was increased up to more than 6.0 (Table 2). It means that the increase in pH by the action of the PGPB would have profound ameliorative effects on rice productivity either in growing medium or in the field.

#### 2.1.4. Effects of Al on the Release of Organic Acids

**Figure 3:**
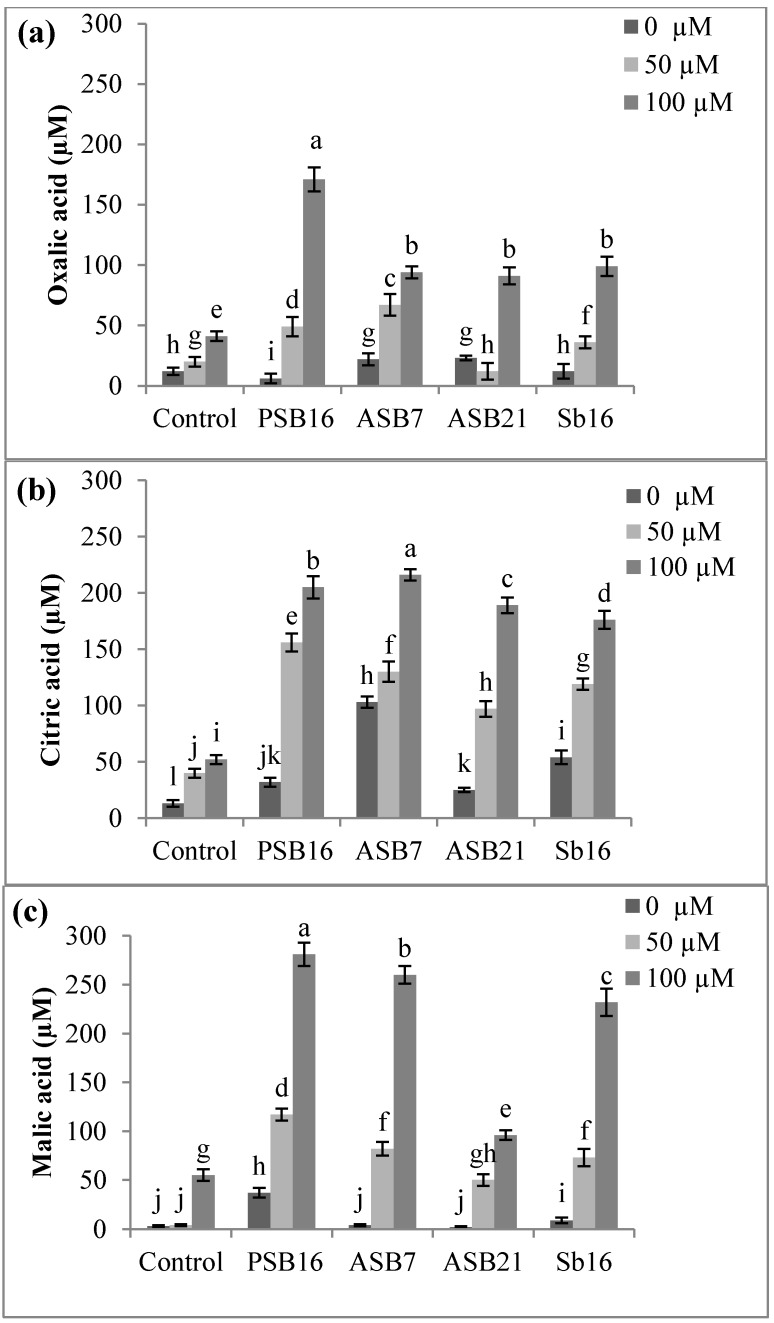
Effects of Al on the release of organic acids by rice plant and or PGPB: (**a**) oxalic; (**b**) citric; and (**c**) malic acid. Where, PSB 16 = *Bacillus* sp., Sb16 = *Stenotrophomonas maltophila*, ASB7 = *Burkholderia thailandensis*, ASB21 = *Burkholderia seminalis.* Means within the same column followed by the same letters are not significantly different at *p* < 0.05. Bars indicate standard error, *n* = 4.

It was observed that organic acids released by the rice roots with or without PGPB varied with Al concentrations (Figure 3a–c). Rice root without PGPB inoculation secreted a lower amount of organic acids compared to those inoculated with the bacteria. The release of organic acids was enhanced by PGPB inoculation at high concentrations of Al. Higher amounts of malic and citric acids were released by the bacteria compared to oxalic acid. The amount of malic acid was found to be high, particularly at 100 µM Al concentration.

#### 2.1.5. Effect of Al on the Rice Roots and Leaf Cells

**Figure 4:**
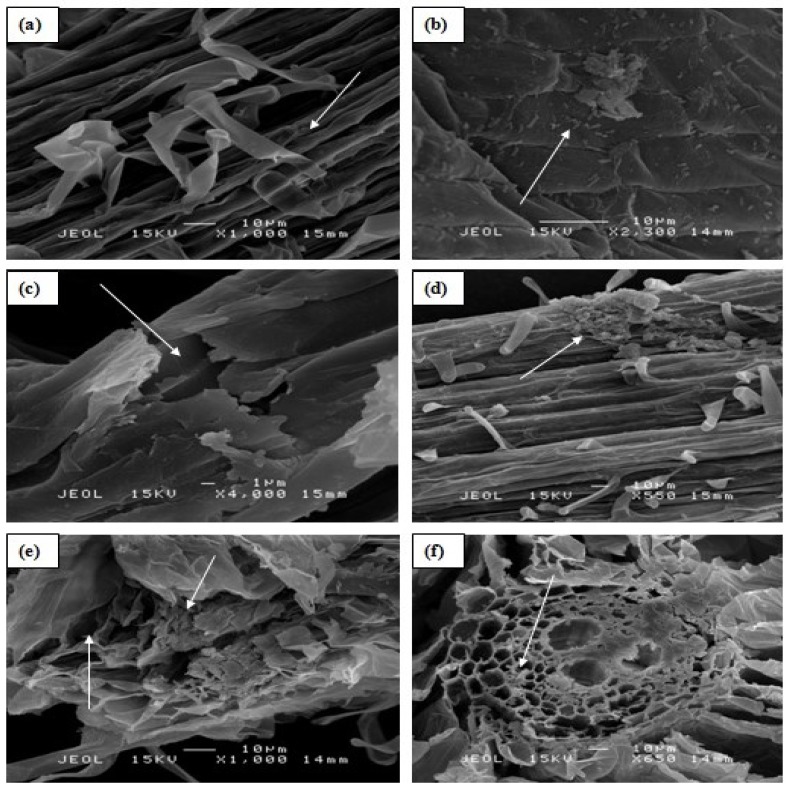
Scanning electron micrographs showing the effects of Al toxicity in rice roots: (**a**) root surface in the control treatment at 0 µM Al, arrow shows no rupturing and microbes presence (**b**) PGPB inoculated root surface at 0 µM Al, arrow shows the microbes on the root surface (**c**) showing root cracks and tearing of the cells in the rhizodermis and outer cortex resulting in the formation of transverse ruptures in the control treatment at 100 µM Al, (**d**) showing PGPB inoculated root surface at 100 µM Al, arrow shows the microbes on the root surface (**e**) showing the effects on the root cortical tissues in the control treatment at 100 µM Al, arrows indicate the distance of a rupture at the interior- and exterior-surface of an individual cell and (**f**) showing root cortical tissues inoculated with PGPB at 100 µM Al concentration, arrow indicates microbes in the root cortical tissues.

Effects of Al on the morphology of rice root cell was visually observed under SEM. High Al concentration (100 µM) affected the root cortical tissues, causing rapture at the root surface, and root cells collapsed in the un-inoculated treatment (Figure 4c,e). The inoculated rice plants still contained bacteria even at 100 µM Al concentration without showing any surface rapture or root cell collapse (Figure 4b,d,f). Cells damage in the root cortex of rice plants without PGPB inoculation was clear evidence of the effects of Al toxicity.

**Figure 5:**
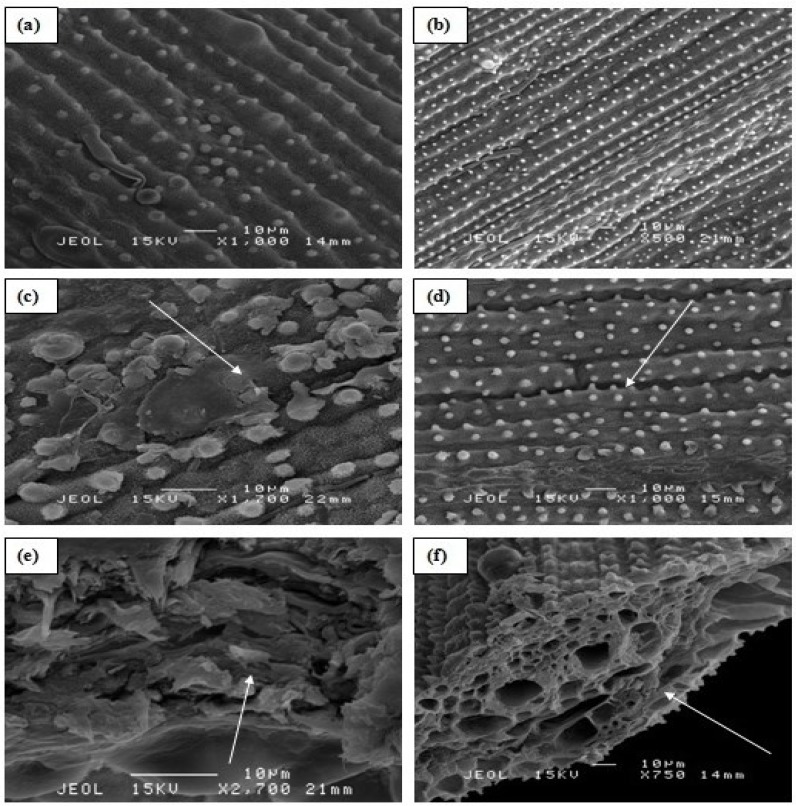
Scanning electron micrographs showing adaxial rice leaf surface and leaf cells: (**a**) showing leaf surface in the control treatment at 0 µM Al; (**b**) showing adaxial leaf surface inoculated with PGPB at 0 µM Al; (**c**) showing ruptured leaf surface in the control treatment at 100 µM Al; (**d**) showing PGPB inoculated leaf surface at 100 µM Al, without any rupturing; (**e**) showing internal destructive leaf cells in the control treatment at 100 µM Al; and (**f**) showing internal healthy leaf cells at 100 µM Al concentration (PGPB inoculated), arrow shows the normal leaf cells.

From the scanning electron microscopic study, it was revealed that at 0 µM Al concentration there were no glands present in the leaf (Figure 5a,b). However, rupture on the rice leaf surface (Figure 5b) and internal leaf cells destruction in the control treatment appeared at 100 µM Al concentration (Figure 5e). The leaf surface damages were observed longitudinally arranged in parallel rows, adjacent to the rows of stomata. There was severe cell rupture occurring at the 100 µM Al concentration (Figure 5e). However, there were no damages (Figure 5d) and cell rapture observed (Figure 5f) in the rice plants inoculated with PGPB even at the highest Al concentration of 100 µM.

### 2.2. Field Study

#### 2.2.1. Effects of Biofertilizer, Basalt and Ground Magnesium Limestone (GML) Applications on the Soil pH in the Rice Field

Application of bio-fertilizer, basalt and GML in the acid sulfate soil increased soil pH from 3.8 to >5.0 and it remained at that level until crop harvest (Table 3). Significantly higher soil pH (5.27) was observed in the biofertilizer and GML treatments compared to that of the others. In basalt treatment, there was only a slight increase in soil pH (4.35) as basalt takes longer time to be completely disintegrated and reacted with the soils.

**Table 3:** Effects of biofertilizer, ground magnesium limestone GML and basalt application on soil pH.

Treatments	Soil pH				
	0	30	60	90	110
	Days after Sowing				
Control	3.8a	3.56f	3.89d	3.95d	3.91d
Biofertilizer	3.8a	4.11d	4.00c	4.14c	4.17c
^a^ GML	3.8a	4.90b	4.39a	4.53b	4.55ab
Basalt	3.8a	3.97e	4.10b	4.46b	4.49b
Biofertilizer + GML	3.8a	5.27a	4.50a	4.76a	4.79a
Biofertilizer + Basalt	3.8a	4.35c	4.20b	4.23c	4.32c

Notes: Means within the same column followed by the same letters are not significantly different at *p* < 0.05 (*n* = 4); ^a^ Ground magnesium limestone.

#### 2.2.2. Effects of Biofertilizer, Basalt and Ground Magnesium Limestone (GML) Applications on the Growth of Rice and Yield

It was noted that aluminum severely affected the growth of rice and eventually its yield. The application of bio-fertilizer in combination with GML and basalt increased the yield of rice significantly. The highest grain yield of 6.82 t·ha^−1^ was obtained for the bio-fertilizer in combination with GML treatment. This is consistent with the highest root length, tiller number, panicle length, fertile spikelets panicle^−1^, filled grains and weight of 1000 grains obtained in this treatment (Table 4).

**Table 4:** Effects of biofertilizer, ground magnesium limestone (GML) and basalt application on the growth and yield of rice.

Treatments	Root Length (cm)	Tillers plant^−1^	Number of Panicle plant^−1^	Size of Panicle ^−1^	Fertile Spikelets panicle^−1^	Number of Unfilled Grains (%)	Weight of 1000 grain (g)	Grain Yield t·ha^−1^	Harvest Index
Control	19.66d	9c	7c	17.83e	61.01e	26.21a	17.02d	2.93d	0.40e
Biofertilizer	32. 41a	20a	14ab	20.10c	119.51b	16.12f	21.40c	5.39b	0.35d
^a^ GML	21.34b	19a	15a	22.60b	116.81b	18.31d	22.33b	5.36b	0.45b
Basalt	20.13c	16b	16a	18.33d	98.85d	20.45b	20.01c	3.47c	0.41c
Biofertilizer + basalt	22.30b	19a	15a	23.00b	107.32c	19.24c	23.68a	5.33c	0.47b
Biofertilizer + GML	32.58a	21a	15a	24.23a	129.03a	17.82e	22.55b	6.82a	0.55a

Notes: Means within the same column followed by the same letters are not significantly different at *p* < 0.05 (*n* = 4); ^a^ Ground magnesium limestone.

#### 2.2.3. Effects of Application of Amendments on the Form of Aluminum in the Soil

Application of amendments on the acid sulfate soil under study had significantly reduced the exchangeable form of Al. The highest exchangeable and weakly-bound Al was found in the control treatment (Table 5). Lower values of exchangeable and weakly-bound Al were found in the bio-fertilizer in combination with GML treatment compared to those of other treatments. However, the highest strongly-bound Al was found in the biofertilizer, while, the lowest was in the control treatment.

**Table 5:** Effects of bio-fertilizer, ground magnesium limestone (GML) and basalt application on the different forms of aluminum in soil after rice harvest.

Amendments (4 t·ha^−1^ each)	Exchangeable Al	Weakly-bound Al	Strongly-bound Al
	cmol_c_ kg^−1^		
Control	2.09a	3.04a	4.78f
Biofertilizer	0.11b	2.03b	11.69a
GML ^a^	0.03d	1.13f	7.23d
Basalt	0.07c	1.65c	9.02b
Biofertilizer + GML	0.01e	1.34e	6.34e
Biofertilizer + basalt	0.04d	1.42d	8.53c

Notes: ^a^ Ground magnesium limestone; Means within the same column followed by the same letters are not significantly different at *p* < 0.05 (*n* = 4).

### 2.3. Discussion

Highly exchangeable Al in acidic soil is a threat to rice growth and yield. In this study, we found that PGPB of the genus *Bacillus*, *Stenotrophomonas* and *Burkholderia* were capable of reducing the effects of Al toxicity. The main mechanism involved was the release of organic acids either by the rice roots or PGPB that chelated Al. Release of organic acids by plant roots can be considered as one of the ways that the plant defends itself against Al toxicity; this phenomenon is probably controlled by the genetic makeup of the rice plant. The reduction of Al toxicity was further enhanced by the presence of PGPB. It was proven that the bacteria were also able to produce not only organic acids, but also phytohormone and exopolysaccharides. The production of phytohormones (IAA) by PGPB has additional benefit for the plant growth and all PGPB were tested and found able to produce phytohormones from the earlier studies. Phytohormone enhanced root architecture that helps increase nutrient uptake from the surroundings. The common organic acids released by the rice roots and bacteria were oxalic, citric and malic acids. Organic acids are known as Al-chelating molecules. The low molecular weight organic acids secreted by plant root chelate Al in the soil solution, forming Al-citrate or Al-malate, preventing it from entering the root cells [15]. The most effective organic acid in alleviating toxic Al and Fe effects was citric, followed by oxalic and tartaric acids. Malic, malonic and salicylic acids were moderate in detoxifying Al and Fe [16].

Plants can detoxify Al in the rhizosphere by producing organic acids which can chelate Al, rendering it unavailable to the growing crops. Citrate and malate are mostly present in the root tips. Under Al stress, rice root exuded organic acids that reduce the effects of Al toxicity by forming Al-citrate or Al-malate. It has been reported that among gramineous crops, rice demonstrates the highest level of Al-tolerance [17].

In the *in vitro* study, we found that high Al concentration severely affected the growth of rice seedlings during the planting period. Rice seedlings inoculated with the PGPB were less affected by the presence of high concentration of Al. The inoculated rice seedlings were shown to perform better compared to that of the non-inoculated seedlings, indicating that the bacteria were able to reduce the effects of Al toxicity via the mechanisms mentioned above.

It was observed in the present study that the PGPB was able to increase pH from 4.0–6.0. The isolates were able to produce polysaccharides that might absorb H^+^ from the solution and increased pH in the rhizosphere zone. This is yet another mechanism that in reducing Al toxicity. At low pH (<5), Al dissolved, causing toxicity to the rice plants [18]. When the pH is increased to a value above 5.0 (the pKa of Al is 5), Al concentration in solution is reduced to a minimal level [19], and hence it is no longer a threat to the growth of rice. In the present study, the increase in pH occurred at the solution-rhizosphere interface. However, when pH was low (<4.8), dissolved Al^3+^ in the soil solution can reach the critical level of 30 µM, which can damage rice plants. Aluminum toxicity is often related to phosphorus deficiency because a soil with high Al concentration will decrease the availability of P due to Al-Fe-phosphate interaction [20]. That is why the most recognized symptom of Al toxicity is P-related inhibition of root growth.

It was observed that bacterial population was slightly affected in the presence of high Al concentration. The microbial population was slightly reduced, most probably due to the increased inhibitory effects by releasing free Al [21], which is consistent with the previous findings [22,23]. The results of our study are in line with those mentioned above. It was proved that PGPB could survive in high Al concentration.

Rice can be grown satisfactorily when Al concentration is <20 µM [24]. Toxicity appears when there is a decrease in mitotic activity as a result of Al exposure to root tips of plants [25]. The inhibition of cell elongation could be the mechanism leading to root growth inhibition [26] as Al^3+^ is attracted to the negatively-charged cell walls of the roots [27]. SEM micrographs of the current study clearly showed the effects of high Al concentration on the root and leaf cells of rice where raptures of root cells and leaf glands were observed. But when the plants were inoculated with PGPB, these features were absent, showing the ameliorative contribution of the bacteria to rice plant.

Al toxicity causes severe changes to root morphology, resulting in curved, swollen, cracked, brownish, stubby and stiff root apices [28]. Al toxicity symptoms in plants are not easily identifiable unless their levels increase [29] which reduces the other nutrients’ availability in the plant. The presence of high concentrations of Al can cause severe damage to the cells of rice roots [30]. The cells could break up and become disjointed, consequently affecting the growth of rice [31]. Liao *et al.* [32] found that non-tolerant Al plants were severely affected by exposure to Al. Aluminum disturbs the growth of cells in the elongation zone of roots [33]. Furthermore, Barker and Pilbeam [34] explained that Al can reduce root cell division; hence, it causes disruption of root cap processes, inhibiting root elongation. We believed that Al can affect rice roots in a similar fashion.

Several microorganisms such as *Sphingomonas* spp. are known to synthesize bacterial exopolysaccharides [35] and have been observed to perform a major role in providing protection to the cell as a boundary layer [36]. It is also able to chelate heavy metals due to the presence of several active functional groups [37].

The presence of high Al concentration severely affects the root morphology and the resulting decrease in root surface area simultaneously causes the Ca and Mg deficiencies [38]. Under this condition, GML application can alleviate the problem. The problem of Al toxicity can somewhat be alleviated by PGPB inoculation by which organic acids are secreted that chelate Al as well as production of phytohormones [12]. The results of the current study are consistent with the above findings. Rice plant has the mechanism to produce organic acids through root exudates, and it was observed in the current study that higher amounts of organic acids were released in the bacterial-inoculated treatments compared to that of the untreated rice seedlings.

In the field study, we found that the application of bio-fertilizer, GML and ground basalt positively affected the growth of rice due to reduction in Al toxicity. The reduction of Al in the soil by the application of GML increased soil pH and supplied sufficient Ca and Mg needed by rice growing in the field. The phosphate-solubilizing bacteria also increased bioavailable P to the plant as P is being fixed in the acidic soil. The presence of the bacteria in the bio-fertilizer helped secrete organic acids that chelated Al in the soil. Treating the acid sulfate soil with bio-fertilizer and GML, either alone or in combination, had alleviated its infertility that resulted in the increase of biomass and eventually rice yield.

It seems that the growth of rice roots was promoted by the bacteria present in the bio-fertilizer. These bacteria have the ability to promote the production of hormones such as indole-3-acetic acid [11] that enhanced plant growth [39,40]. Furthermore, bacterial inoculation ensured significant yield improvement by increasing P uptake, supplying N_2_ and phytohormone production.

The analysis of the soil showed significantly higher amounts of weakly-bound Al present in the control treatment, but in the bio-fertilizer or GML treatment, its amount was lowered significantly. This phenomenon had proven beyond doubt the possible chelation of the free Al in acid sulfate soils by the organic acids produced by the bacteria or a chemical reaction resulting from GML application. The presence of lower amounts of weakly-bound Al in the soil had promoted rice root development and increased surface area for higher nutrient uptake, leading to the increase in rice yield [41,42].

## 3. Experimental Section

### 3.1. Experimental Site/Preparation and Conditions

Laboratory experiments were conducted at Universiti Putra Malaysia, Serdang, while the field study on acid sulfate soil was conducted at Semerak, Kelantan, Malaysia. The main objective of laboratory study was to determine the mechanism of plant PGPB to alleviate Al toxicity and growth of rice. The PGBP isolates used were *Bacillus* sp. PSB16 Accession No: JX103827 (phosphate-solubilizing bacteria), *Stenotrophomonas maltophila* Sb16 Accession No: NR 041577.1 (Nitrogen fixing bacteria), *Burkholderia thailandensis*ASB7 Accession No: NR 074312.1 (acid sulfate tolerant bacteria) and *Burkholderia seminalis* ASB21 Accession No: NR 042635.1 (acid sulfate tolerant bacteria). The ASB7 and ASB21 were isolated from the acid sulfate rice soils in Semerak, Malaysia, while the PSB16 and Sb16 were isolated from other rice fields in Malaysia. In the field study, the effect of PGPB present in bio-fertilizer, GML and ground basalt were studied. High yielding rice variety, MR219 was grown on an identified acid sulfate soil. Soil pH, total N, organic C, available P, exchangeable K, exchangeable Al, exchangeable Ca and exchangeable Mg was 3.80, 0.12%, 2.10%, 19.20 mg·kg^−1^, 0.05, 4.30, 0.60 and 0.70 cmol_c_ kg^−1^, respectively.

### 3.2. Laboratory Study

Rice seedlings (MR219) were inoculated with washed bacterial inoculums at approximately 5 × 10^9^ CFU·mL^−1^. Plant seedlings were grown for 21 days in growth chamber with 12 h light/dark cycle at 29 ± 1 °C. The experiment was arranged in a completely randomized design (CRD) with four replications.

#### 3.2.1. Preparation of Inocula and Rice Seedlings Inoculation under *in vitro* Condition

The bacterial strains were cultured in specific media plates for purity. The PSB were cultured in National Botanical Research Institute for Phosphorus agar NBRIP while N_2_ fixing bacteria were cultured in Jenson’s broth for 72 h. At the exponential growth stage, cells were harvested by centrifugation and washed with phosphate buffer solution. Approximately 5 × 10^9^ mL^−1^ of live washed bacterial cells were used as inoculums in each bacterial treatment after transplanting. The population was confirmed by cell enumeration in drop plate method on NBRIP agar plate [43]. The rice seeds (MR219) were surface-sterilized [44]. The seeds were sown in a plastic tray lined with moist filter paper. Sterilized distilled water was added daily to moisten the seeds. Seedlings were grown for 7 days, after which three seedlings were transferred into Hoagland solution containing different concentrations of Al (0, 50 and 100 µM) and grown for 21 days. The initial pH of the solution was adjusted to 4.0. Bacterial population, plant dry biomass, solution pH, organic acids were determined. Root morphology was viewed under scanning electron microscope.

#### 3.2.2. Determination of Microbial Population at Different Al Concentrations

One mL of broth was taken from Al treated sample at the initial and end of the study periods (21 days) for determination of bacterial growth. A series of 10-fold dilution were prepared up to 10^−10^. The population of the bacteria was determined using drop plate count method on NBRIP media plate [43].

#### 3.2.3. Determination of Organic Acids and Indoleacetic Acid

Organic acids were determined from plant growth medium at harvest using high performance liquid chromatography (HPLC) (Jasco Borwin software). Each sample from each treatment was injected into HPLC with a UV detector set at 210 nm, using a Rezex ROA-organic acid H^+^ (8%) column (250 × 4.6 mm) from Phenomenex Co. (Torrance, CA, USA); 0.005 N H_2_SO_4_ was used as mobile phase with a flow rate of 0.17 mL·min^−1^. The amount of organic acids produced by the PGPB were estimated by deducting the organic acids present in the plant growth medium of control treatment from those present in the bacterial applied treated samples.

The PGPB were inoculated in broth with addition of tryptophan (2 mg·L^−1^) and incubated at 28 ± 2 °C for 48 h. The culture was centrifuged at 7000 rpm for 7 min and 1 mL of the supernatant was mixed with 2 mL of Salkowsky’s reagent [45]. The indoleacetic acid (IAA) was determined using spectrophotometer at 535 nm.

#### 3.2.4. Determination of Root Morphology

The morphology of the rice roots was studied by a root scanner (model Epson Expression 1680, equipped with root scanning analysis software). Total volume (cm^3^) was quantified using the scanner [46]. The scanned roots data were processed by Win-Rhizo© software (Reagent Instruments Inc., Québec, QC, Canada).

#### 3.2.5. Visual Observation of Plant Leaf and Root Cells

Plant leaf and root cells of rice seedlings with bacterial colonization were observed under scanning electron microscope (SEM). The leaves and roots were cut into 1 cm and were pre-fixed with 4% glutaraldehyde overnight and washed with 0.1 M sodium cacodylate buffer 3 times for 30 min each. Osmium tetraoxide buffer (1%) was used for post fixation. After a series of dehydration in acetone (35%, 50%, 75%, 95% and 100%), the samples were dried in a critical point dryer and mounted on aluminum stubs, sputter-coated with gold and viewed under SEM (JEOL JSM-6400 attached with OXFORD INCA ENERGY 200 EDX).

### 3.3. Field Study

#### 3.3.1. Bio-Fertilizer, GML and Basalt Application and Transplanting

The field study comprised six treatments: (i) Control [without any soil amendment]; (ii) bio-fertilizer at 4 t·ha^−1^; (iii) GML 4 t·ha^−1^; (iv) basalt 4 t·ha^−1^; (v) bio-fertilizer + GML (4 t·ha^−1^ each); and (vi) bio-fertilizer + basalt (4 t·ha^−1^ each). The bio-fertilizer is the consortium of four isolates *Bacillus* sp. PSB16 Accession No: JX103827 (phosphate-solubilizing bacteria), *Stenotrophomonas maltophila*Sb16 Accession No: NR 041577.1 (nitrogen fixing bacteria), *Burkholderia thailandensis*ASB7 Accession No: NR 074312.1 (acid sulfate tolerant bacteria) and *Burkholderia seminalis*ASB21 Accession No: NR 042635.1 (acid sulfate tolerant bacteria) with the empty fresh bunch of oil palm and peat (1:1) contained 1.2% N, 0.12% P, 0.65% K, and 48% C. Approximately 5 × 10^9^ g^−1^ of bacterial cells were used in the carrier material of biofertilizer. Twenty-one-day old rice seedlings (MR219) were transplanted on the research plots. The amendments (GML and ground basalt) were mixed thoroughly according to the treatments into the soil 15 days before transplanting. Nitrogen, phosphorus and potassium in the form of urea, rock phosphate and muriate of potash (KCl) were applied at 120, 30 and 60 kg·ha^−1^, respectively. Bio-fertilizer was formulated using the bacterial strains and was applied in the rice field planting and mixed thoroughly into the soil. Each plot size was 5 × 5 m^2^ and was arranged in a Randomized Completely Block Design (RCBD) with four replications.

#### 3.3.2. Speciation of the Al in the Soils

The Al forms in the soils were sequentially extracted by the following procedures: (i) exchangeable Al was extracted with 1M KCl at 1:10 (soil/solution ratio) by shaking for 24 h; (ii) weakly organically-bound Al form was extracted with 0.3 M CuCl_2_ at 1:10 (soil to solution ratio) by shaking for 2 h; and (iii) total organically-bound Al form was extracted with 0.1 M Na_4_P_2_O_7_ at 1:10 (soil/solution ratio) by shaking for 24 h. In all steps, the supernatant was separated by centrifugation for 20 min at 13,500 rpm. The quantity of strongly organically-bound Al was calculated as the difference between Na_4_P_2_O_7_ extracted-Al and CuCl_2_ extracted-Al [47]. The Al in the solution was analyzed using inductively coupled plasma atomic emission spectroscopy (ICP-AES).

#### 3.3.3. Rice Yield and Yield Contributing Characters

Rice plant was harvested at grain maturity. At harvest, agronomic parameters (plant height, tiller number, root length) grain and straw yield were determined according to the procedure detailed [48].

#### 3.3.4. Statistical Analysis

All data were statistically analyzed using the SAS Software Program (Version 9.3), and treatment means were separated by using Tukey’s test (*p* < 0.05).

## 4. Conclusions

Aluminum toxicity is a common problem reducing the yield of rice grown in acid sulfate soils. This problem was alleviated by application of PGPB. The mechanism involved in the ameliorative process was chelation of free Al by the organic acids produced by the bacteria. These bacteria also increased soil pH that precipitated Al and produced phytohormone; both phenomena enhanced rice growth. Scanning electron micrographs of the roots and leaf tissues showed the clear ameliorative effects of PGPB inoculation. Furthermore, rice by itself was able to secrete organic acids via its roots when it was under the stress of high Al concentration. For the rice growing in the field, application of bio-fertilizer containing PGPB had increased yield due to the reasons mentioned above. Rice yield can also be increased further by applying GML or basalt at the appropriate rate. Low exchangeable and weakly-bound Al observed in the bio-fertilizer treatment were clear evidences for the chelation of Al by the organic acids. Hence, the PGPB under investigation can be used for the production of bio-fertilizer for rice cultivation in acid sulfate soils.

## Abbreviations

GML: ground magnesium basalt
HPLC: high performance liquid chromatography
PGPB: Plant growth promoting bacteria
PSB: phosphate solubilizing bacteria
SEM: scanning electron microscope
